# AI-based protein models enhance the accuracy of experimentally determined protein crystal structures

**DOI:** 10.3389/fmolb.2023.1208810

**Published:** 2023-06-22

**Authors:** Ki Hyun Nam

**Affiliations:** ^1^ Department of Life Science, Pohang University of Science and Technology, Pohang, Republic of Korea; ^2^ POSTECH Biotech Center, Pohang University of Science and Technology, Pohang, Republic of Korea

**Keywords:** protein structure, re-refinement, artificial intelligence, AlphaFold, model structure

## 1 Introduction

Structural biology techniques aid the intuitive comprehension of biomolecules by elucidating the underlying molecular mechanisms (Curry, 2015). The RCSB Protein Data Bank (PDB) is an example of a repository comprising over 200,000 macromolecular three-dimensional (3D) structures. These structures are experimentally determined using techniques such as X-ray crystallography, nuclear magnetic resonance (NMR), cryo-electron microscopy (cryo-EM), neutron crystallography, and microcrystal electron diffraction (MicroED) (Bittrich et al., 2023). These structures are crucial for understanding the molecular basis of biomolecule functions. Moreover, they may also provide insights into novel drug designs and the rational engineering of pharmaceutically important antibodies and enzymes (Bornscheuer et al., 2012; Hummer et al., 2022). Therefore, the precision of the biomolecular structures determined by structural biology is essential as it directly influences subsequent research employing the structures constituting the PDB.

The accuracy of the experimentally determined structures may be influenced by the quality of the electron density map in terms of resolution, radiation damage, and molecular flexibility and the researchers’ molecular modeling ability (Palamini et al., 2016; Casañal et al., 2020; Thompson et al., 2020; Shelley and Garman, 2022). High-resolution structural data can provide a distinct electron density map that may precisely detect the positions of atoms within biomolecules (Blakeley et al., 2015). Ill-defined electron density maps with disordered regions attributable to molecular flexibility may result in quality discrepancies. When the electron density map is ambiguous, the model may contain unintentional structural errors. In some cases, molecular modeling may be ignored, leaving the positive Fo-Fc electron density map as it is and depositing the coordinates to PDB.

One method for constructing a model structure based on an experimentally ambiguous electron density map involves tracing the main chain in compliance with previously reported topology or homolog structural folding (Lance et al., 2010; Hameduh et al., 2020). Meanwhile, in the absence of a reference model structure, the most recent artificial intelligence (AI)-generated model may be used (Hameduh et al., 2020).

Advances in AI technology have facilitated pioneering research in structural biology with highly accurate 3D protein structure prediction algorithms such as AlphaFold2 (Jumper et al., 2021) and RoseTTAfold (Baek et al., 2021). In particular, DeepMind, a Google AI derivative, has achieved marked progress in biology with the AI network Alphafold2 to accurately predict the 3D structure of proteins (Callaway, 2020). Alphafold2 produces accurate models with an estimated precision of less than 1Å for the position of both the backbone and sidechains of protein. The RCSB PDB now provides access to over one million protein-calculated structure models (CSMs) through the AlphaFold DB and ModelArchive (Bittrich et al., 2023). Information on formerly inaccessible structures can now be readily obtained without specialized programming. AI-generated structures are widely used as search models for molecular replacement in X-ray crystallography (Nam et al., 2022). They are also used as initial models in cryo-EM (Hu et al., 2022a; Hu et al., 2022b). Therefore, AI-based molecular modeling has contributed revolutionarily to the determination of 3D experimental structures.

Conversely, older structures deposited in the PDB frequently exhibit poor structural quality. This may be due to the lack of precision compared to those engineered using modern molecular modeling or crystallographic structure refinement software (Emsley and Cowtan, 2004; Winn et al., 2011; Liebschner et al., 2019). PDB-REDO, which combines refinement and rebuilding within a unique decision-making framework, solves this by giving researchers access to more accurate structural information through models that adhere to recent crystallographic standards (Joosten et al., 2009; Joosten et al., 2012; Joosten et al., 2014). However, the PDB also contains models with ambiguous electron densities in which the amino acid positions are indistinct, requiring more precise structural models. Moreover, some experimentally determined structures have precision (accurate data, high resolution) but may be inaccurate due to errors in the main chain tracing of protein.

This study demonstrates that the imprecise molecular locations in an ambiguous electron density map of the PDB-deposited structural model can be improved using AI-predicted model structures. Employing these AI model structures in conjunction with modern crystallographic techniques will increase the precision of experimentally determined structures. This will also contribute to the advancement of fundamental scientific applications.

## 2 Improvement of experimentally determined model structure by Al model structure

To suggest the possibility of existing model enhancement by referencing the Al models, two previously deposited model structures (PDB codes 1JHN and 2Z1B) lacking certain amino acid sequences were chosen from the PDB. AI model structures were retrieved from the AlphaFold DB (https://alphafold.ebi.ac.uk/). The AI-predicted structures were superimposed on the selected model structures to verify model improvement and validate that the orientations of the side and main chains of the proteins were reasonable.

### 2.1 Improvement of the lumenal domain structure of calnexin

**FIGURE 1:**
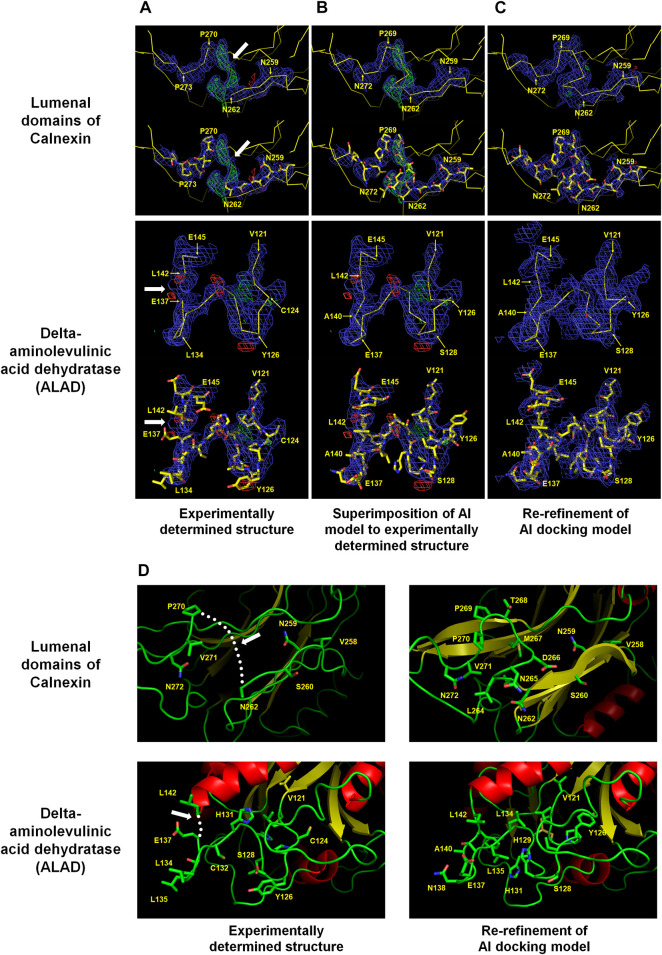
Improvement of experimentally determined the lumenal domains of Calnexin (PDB code: 1JHN) and Delta-aminolevulinic acid dehydratase (ALAD) (PDB code: 2Z1B) by using the AI system AlphaFold. The regions Asn262–Pro270 of the lumenal domain and Val121–Leu142 of ALAD were reconstructed using the AI model structure. **(A)** Experimentally determined structures **(B)** Superimposition of the AI models on the experimentally determined model structures **(C)** COOT program-based real-space refinement of the Al model structure followed by a subsequent re-refinement using the phenix.refine software. The 2Fo-Fc (blue, 1 σ) and Fo-Fc (green for 3 σ and red for −3 σ) electron density maps are illustrated in mesh. **(D)** Cartoon representation of the experimentally determined and improved model structures in accordance with the AI model structure. The regions where amino acid model building was not performed are indicated by white arrows or dotted lines.

Calnexin is an endoplasmic reticulum (ER)-associated type I integral membrane protein that belongs to a family of molecular chaperones. The lumenal domain of calnexin interacts with the nascent chain of newly synthesized N-linked glycoproteins upon entry into the ER lumen and facilitates productive protein folding and assembly (Ou et al., 1995). The crystal structure of the lumenal domain of calnexin (PDB code: 1JHN) was determined at 3.1 Å resolution using three independent phase sets derived from a combination of isomorphous replacement and anomalous scattering phasing techniques. The lumenal domain consists of a compact globular domain comprising a β sandwich of two antiparallel β-sheets and a long arm stretched away from the globular domain. A positive Fo-Fc electron density map was observed between Asn262 and Pro270 in the globular domain (Figure 1A). In terms of electron density, the positive Fo-Fc electron density map barely has space to construct seven amino acids between Asn262 and Pro270.

The amino acids were estimated to have been compacted at the positions corresponding to the N- and C-terminals of Asn262 and Pro270, respectively. However, it was difficult to accurately define the position of C-alpha due to the indistinct electron density map. The AlphaFold model was referenced to define the locations of the disconnected amino acids between Asn262 and Pro270 (average pLDDT: 91.88) in the global lumenal domain model structure. The C-alpha chain fitted very well into the disconnected area by superimposing the model structure on the experimental globular domain in the Coot program (Figure 1B). Consequently, re-refinement by replacing the Asn262 and Pro270 regions in the existing model with an Alphafold structure validated the greater reliability compared with that of the previous model (Figure 1C). Conversely, the accuracy of the side chain position was uncertain due to the quality of the electron density map. Instead, it could be concluded that, based on the model structure, the hydrophilic and hydrophobic amino acid orientations were reasonably accurate (Figure 1D).

### 2.2 Improvement of delta-aminolevulinic acid dehydratase structure

Delta-aminolevulinic acid dehydratase (ALAD) catalyzes the second step of the heme biosynthesis pathway. This involves the condensation of two molecules of delta-aminolevulinic acid (δ-ALA) into porphobilinogen (Liu et al., 2020). Pb can disrupt cellular structures, damage the cell membrane, and impede DNA transcription (Collin et al., 2022). ALAD is highly sensitive to divalent Pb ions, rendering it a valuable indirect biomarker for estimating Pb exposure in humans (La-Llave-León et al., 2017). Two crystal structures of *Mus musculus* 5-ALA dehydratase were deposited under the PDB codes 2Z0I (selenomethionine derivatives, 3.2 Å) and 2Z1B (native, 3.3 Å) (unpublished). These proteins possess α+β folds with flexible N-terminal domains and several disordered loop regions. The electron density maps between the 125th and 140th amino acids of ALAD were not clearly observed. However, in the B chain of native ALAD, this region exhibited an electron density map, and the model structure was constructed, except between amino acids Glu137 and Leu142. The B chain of ALAD required the construction of 5 amino acids between Glu137 and Leu142, but the space available on the electron density map was insufficient (Figure 1A). This was a construction error based on the ambiguity of the electron density map during molecular modeling. The AlphaFold-generated predicted model structure was superimposed on the B chain of native ALAD to verify whether it could be improved using an AI model. The AlphaFold model (Val121–Leu142: average pLDDT >95.67) demonstrated a highly accurate fit on the electron density map (Figure 1B). This confirmed that shifting the main chains of the B chain of native ALAD from Cys124 to Glu137 rendered the space between Glu137 and Leu142 to be insufficient, with a rational orientation of all amino acid side chains. The re-refinement result of the B-chain of native ALAD indicated a reliable model structure without a positive or negative Fo-Fc electron density map (Figure 1C). Therefore, an AI structure may be used as a reference for the creation of a reliable model structure from a previously flawed ALAD model (Figure 1D).

## 3 Discussion

PDB users employ experimentally determined structures to comprehend molecular mechanisms and design experiments applicable in various fields, including rational protein engineering or inhibitor designing for novel drug design. Numerous biological studies have employed experimentally determined PDB structures to elucidate their findings. Accordingly, an accurate and reliable experimentally determined 3D structure is essential for supporting current and future research. The resolution, R-free value, and validation report are excellent criteria for evaluating model structures, as indicated in several incisive studies. However, this information does not constrain the accuracy of the PDB model structures. To date, all coordinates and structure factors have been deposited in the PDB; consequently, structural biologists can validate experimentally determined coordinates using an electron density map and assess the precision of the model structure. However, it is difficult for researchers lacking structural expertise to find building errors in PDB model structures, which can subsequently result in catastrophically negative conclusions in future studies. Therefore, for credible results in subsequent research, the experimental model structures must be improved to precise structures with the modern crystallography software or a reliable model structure for main-chain tracing. In this respect, it is crucial to enhance the experimentally determined structures deposited in the PDB using cutting-edge technology. This study demonstrated the enhancement of the experimentally ambiguous parts of the structures (PDB codes 1JHN and 2Z1B) using an AI model structure. This reinforces the significance of AI in increasing the efficiency of subsequent studies through precise improvement of existing experimental results. The use of AI models to improve the existing experimental structures yielded the following empirical findings: 1) The experimental results for the protein-folding components were highly consistent with those predicted by the AI model. 2) The linker region, where the protein is flexible, has a relatively low concordance and requires further model refinement. 3) For proteins with two or more domains, the experimental results and the prediction model may differ regarding the conformation of the two domains. 4) The AI model cannot be referenced when the quality of the electron density in the experimental data is inaccurate or disordered. These findings may be helpful in contributing to the improvement of other experimentally determined structures using AI models. Meanwhile, structural differences may arise between the experimentally determined crystal structures and the AI model structures. In crystallographic structures, the conformations of loops are often affected by crystal contacts, which may not correspond to the conformation in solution as determined by NMR (Laurents, 2022). As a result, the accuracy of the Alphafold2 model may be subject to bias and limitations. However, Alphafold2 generates metrics such as PAE and pLDDT, which provide valuable information about the model’s quality. By considering these metrics, one can objectively evaluate the strengths and limitations of AI predictive models.

In conclusion, the AI-based model structures can improve the experimentally determined PDB structures. However, the wider utility of this theory for all existing structures requires further research. Based on the experimental findings, it is evident that the use of AI models for ambiguous electron-density maps improves model structures. Experimental results can be improved when the experimental and AI structures are used complementarily. The determination of more structures and continuous advancements in AI will generate more precise structural data. Consequently, with the future provision of a platform for the deposition of AI-based improved models, the use of PDB structures may facilitate more credible and convincing follow-up studies.
